# Whole blood transcriptional profiles as a prognostic tool in complete and incomplete Kawasaki Disease

**DOI:** 10.1371/journal.pone.0197858

**Published:** 2018-05-29

**Authors:** Preeti Jaggi, Asuncion Mejias, Zhaohui Xu, Han Yin, Melissa Moore-Clingenpeel, Bennett Smith, Jane C. Burns, Adriana H. Tremoulet, Alejandro Jordan-Villegas, Damien Chaussabel, Karen Texter, Virginia Pascual, Octavio Ramilo

**Affiliations:** 1 Division of Pediatric Infectious Disease, Nationwide Children’s Hospital, Columbus, OH, United States of America; 2 Center for Vaccines and Immunity, The Research Institute at Nationwide Children’s Hospital, The Ohio State University College of Medicine, Columbus, OH, United States of America; 3 Baylor Institute for Immunology Research, Dallas, TX, United States of America; 4 Center for Biostatistics, The Research Institute at Nationwide Children’s Hospital Columbus, OH, United States of America; 5 Department of Pediatrics, University of California San Diego and Rady Children’s Hospital, San Diego, CA, United States of America; 6 Division of Pediatric Infectious Diseases, University of Texas Southwestern Medical Center, Dallas, TX, United States of America; 7 Sidra Medical and Research Center, Doha, Qatar; 8 Division of Pediatric Cardiology, Nationwide Children’s Hospital, Columbus, OH, United States of America; 9 Drukier Institute for Children’s Health, and Weill Cornell Medicine, New York City, NY, United States of America; Institut National de la Santeet de la Recherche Medicale (INSERM), FRANCE

## Abstract

**Background:**

Early identification of children with Kawasaki Disease (KD) is key for timely initiation of intravenous immunoglobulin (IVIG) therapy. However, the diagnosis of the disease remains challenging, especially in children with an incomplete presentation (inKD). Moreover, we currently lack objective tools for identification of non-response (NR) to IVIG.

**Methods:**

Children with KD were enrolled and samples obtained before IVIG treatment and sequentially at 24 h and 4–6 weeks post-IVIG in a subset of patients. We also enrolled children with other febrile illnesses [adenovirus (AdV); group A streptococcus (GAS)] and healthy controls (HC) for comparative analyses. Blood transcriptional profiles were analyzed to define: a) the cKD and inKD biosignature, b) compare the KD signature with other febrile illnesses and, c) identify biomarkers predictive of clinical outcomes.

**Results:**

We identified a cKD biosignature (n = 39; HC, n = 16) that was validated in two additional cohorts of children with cKD (n = 37; HC, n = 20) and inKD (n = 13; HC, n = 8) and was characterized by overexpression of inflammation, platelets, apoptosis and neutrophil genes, and underexpression of T and NK cell genes. Classifier genes discriminated KD from adenovirus with higher sensitivity and specificity (92% and 100%, respectively) than for GAS (75% and 87%, respectively). We identified a genomic score (MDTH) that was higher at baseline in IVIG-NR [median 12,290 vs. 5,572 in responders, p = 0.009] and independently predicted IVIG-NR.

**Conclusion:**

A reproducible biosignature from KD patients was identified, and was similar in children with cKD and inKD. A genomic score allowed early identification of children at higher risk for non-response to IVIG.

## Introduction

Kawasaki disease (KD) is a febrile vasculitis of unknown etiology that affects young children. The estimated annual incidence is 17–21 per 100,000 children under the age of 5 in the United States [1–3]. Studies have shown that treatment within the first ten days of illness with intravenous immunoglobulin (IVIG) and aspirin significantly reduces the incidence of coronary artery abnormalities (CAA) [4]. However, the diagnosis of KD is challenging, especially for children whose presentation lack the full clinical spectrum, termed as “incomplete” KD. A delay or lack of diagnosis of both complete and incomplete KD may have long-term consequences [5].

Children with KD that develop persistent or recrudescent fever after IVIG are at higher risk of developing CAA. In Japan, the application of clinical scoring systems to identify children at higher risk for treatment failure and thus developing CAA, has proven useful to intensify primary treatment with IVIG with adjunctive treatments such as corticosteroids [6]. However, in the multi-ethnic, non-Japanese population such as that in the United States, identification of children at high-risk for non-response to IVIG and/or development of CAA has been difficult using clinical criteria alone.

Previous studies have demonstrated the value of gene expression profiling to aid in the assessment of disease severity in children with infectious or autoimmune diseases, and to differentiate KD from other mimicking conditions [7–15]. The major goals of this study were to utilize gene expression profiling: 1) to define a KD transcriptional biosignature that can aid in the characterization of complete and incomplete KD in children, 2) to define the specificity of the KD biosignature compared with that from children with other febrile illnesses such as adenovirus and Group A streptococcus infections (GAS), and 3) to assess the value of a genomic score [molecular distance to health (MDTH)] assessed before IVIG treatment to determine the likelihood of non-response to IVIG therapy.

## Materials and methods

### Patient characteristics

From June 2007 to March 2013, we prospectively enrolled and obtained blood samples in a total of 162 children < 18 years of age; 125 children hospitalized with KD or other febrile illnesses and 37 healthy, asymptomatic children as controls who were age-, gender- and race-matched. Of the 125 hospitalized children, 89 meet the definition of KD [76 met criteria for complete KD and 13 for incomplete KD based on the American Heart Association (AHA) criteria [5], 19 patients had confirmed adenovirus infection, and 17 children had GAS infection. All children with KD were enrolled before IVIG treatment and sequential samples collected at 24 h and 4–6 weeks post-IVIG in a subset of KD patients. Healthy controls were enrolled when undergoing elective surgery for non-infectious related causes, or at routine outpatient visits.

Study participants were enrolled, as a convenience sample, at three hospitals: Nationwide Children’s Hospital in Columbus, OH, Children’s Medical Center in Dallas, TX and Rady Children’s Hospital in San Diego, CA. The Institutional Review Boards (IRB) from these institutions approved the study and written informed consent was obtained from legal guardians.

#### Study design and definitions

**Fig 1:**
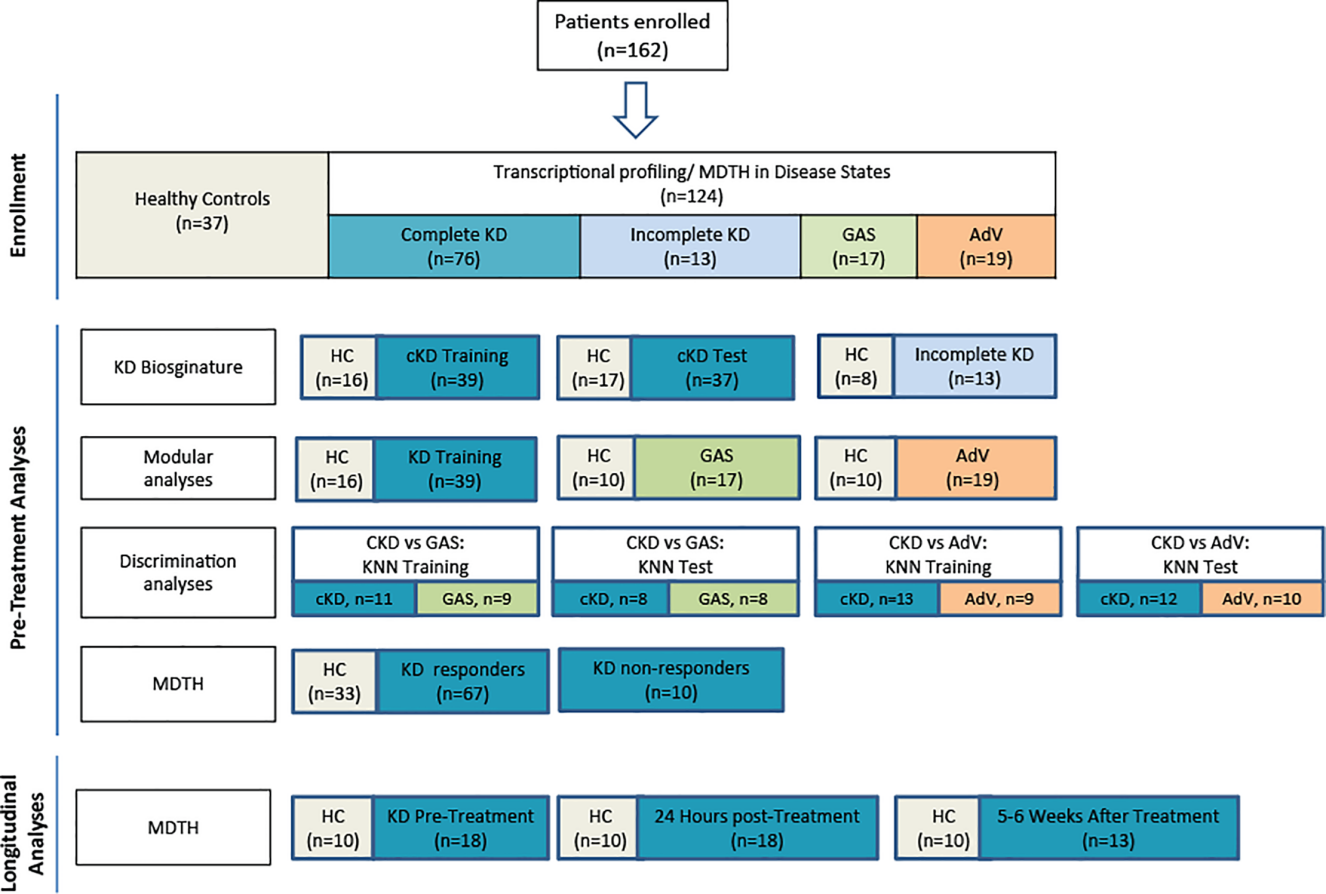
Flow diagram of study patients. Patient allocation and analyses performed throughout the study is depicted in Fig 1. Patients included in the different sub-analyses were matched for age, gender and race/ethnicity with controls.

To define the KD signature, KD patients were divided into three groups: a training, a validation set (test set) that included children with complete KD, and a third set of children with incomplete KD. To determine the specificity of the KD signature, KD transcriptional profiles were compared with those of children with GAS and adenovirus infections. Finally, to determine if the transcriptional genomic perturbation assessed by the molecular distance to health (MDTH) genomic score could predict clinical outcomes, we calculated the MDTH score from KD patients pre-IVIG treatment and assessed its ability to predict the response to IVIG and the development of CAA, after adjusting for other variables (Fig 1). We also assessed changes in MDTH, in children with KD in whom sequential samples were available. Coronary artery abnormalities (CAA) were defined as a Z score ≥2.5 for either the right coronary artery or left anterior descending at any time point, and IVIG resistance as persistent or recrudescent fever at least 36 hours after completion of IVIG treatment.

### Sample collection

Blood samples (1–3 mL) were collected in Tempus tubes (Applied Biosystems, CA, USA) before IVIG treatment in all KD patients, or at diagnosis in children with adenovirus and GAS infections. All samples were stored at -20°C until further processing in batches. Whole blood RNA was processed and hybridized into Illumina Human HT12 V4 beadchips (47,323 probes) and scanned on the Illumina Beadstation 500 as described [9, 14]. The data is deposited in the NCBI Gene Expression Omnibus (GEO accession number: GSE68004).

### Microarray data and statistical analysis

Illumina GenomeStudio software was used to subtract background and scale average samples’ signal intensity and GeneSpring GX 7.3 software was used to perform further normalizations and analyses [14, 16, 17]. Briefly, transcripts were first selected if they were present in ≥ 10% of all samples and had a minimum of 2-fold expression change compared with the median intensity across all samples [14]. Using this approach a total of 11,096 quality control transcripts was obtained. We then followed the strategy outlined below [13]: **a)** *Supervised analysis* (comparative analyses between predefined sample groups) was performed using Mann-Whitney (p< 0.01), followed by Benjamini-Hochberg multiple test corrections and a ≥ 1.25 fold change filter in expression level relative to the control group; **b)** *Unsupervised clustering* (unbiased grouping of samples based on their molecular profile without prior knowledge of sample classification) was applied to the test and validation sets; **c)** *Class prediction* using the K-Nearest Neighbors (KNN), with six neighbors and a P value ratio cutoff of 0.5, was applied to identify the top ranked genes that best discriminated between KD, adenovirus and GAS infections in the training and test sets. We, then, calculated the sensitivity and specificity of the top classifier transcripts in those test sets [13, 14, 16, 18]; **d)** *Functional gene analyses* were performed using modular analysis as described [9, 14, 19]. Module transcript content and annotations are at http://www.biir.net/public_wikis/module_annotation/V2_Trial_8_Modules, and, **e)** *Molecular distance to health (MDTH)*, a tool that converts the global transcriptional perturbation of each sample into an objective score compared with the healthy controls as reference, was calculated and correlated with parameters of disease severity. To calculate the MDTH scores we used the dispersion of expression values found in the baseline samples (controls) to determine whether the expression values of a patient’s given sample lay outside two standard deviations (± 2SD) of the mean value for the healthy controls. Thus, each individual score represents the overall transcriptional perturbation (“distance”) per patient sample in relation to the healthy control baseline. By summarizing the overall transcriptional activity in one score, the MDTH facilitates correlation with clinical parameters and has previously been applied in other disease states [9, 14, 20].

Univariate followed by multivariable logistic regression analyses were conducted to identify which factors independently predicted IVIG resistance, the primary outcome. Although the study was not adequately powered for this purpose, as an exploratory objective, we also examined if MDTH could predict the development of coronary artery abnormalities (CAA, either coronary aneurysm or ectasia). We first calculated the area under the receiver operating characteristic (ROC) curve (AUC) using the pre-treatment MDTH values to determine its ability to discriminate responders versus non-responders to IVIG. The optimal threshold for MDTH was defined using Youden’s J statistic, which maximizes sensitivity and specificity [21]. For multivariable analyses, covariates were included in the model if they were significant in univariate analysis or clinically relevant. Predictor variables with a p <0.05 and multivariate odds ratios (OR) and 95% confidence intervals that did not include 1 were considered significant. Statistical analyses were performed using, Graph Pad Prism V5 (San Diego, CA) and SAS 9.3 (SAS Institute, Inc., Cary, NC).

## Results

### Kawasaki Disease transcriptional signature is reproducible in children with both complete and incomplete presentation

**Fig 2:**
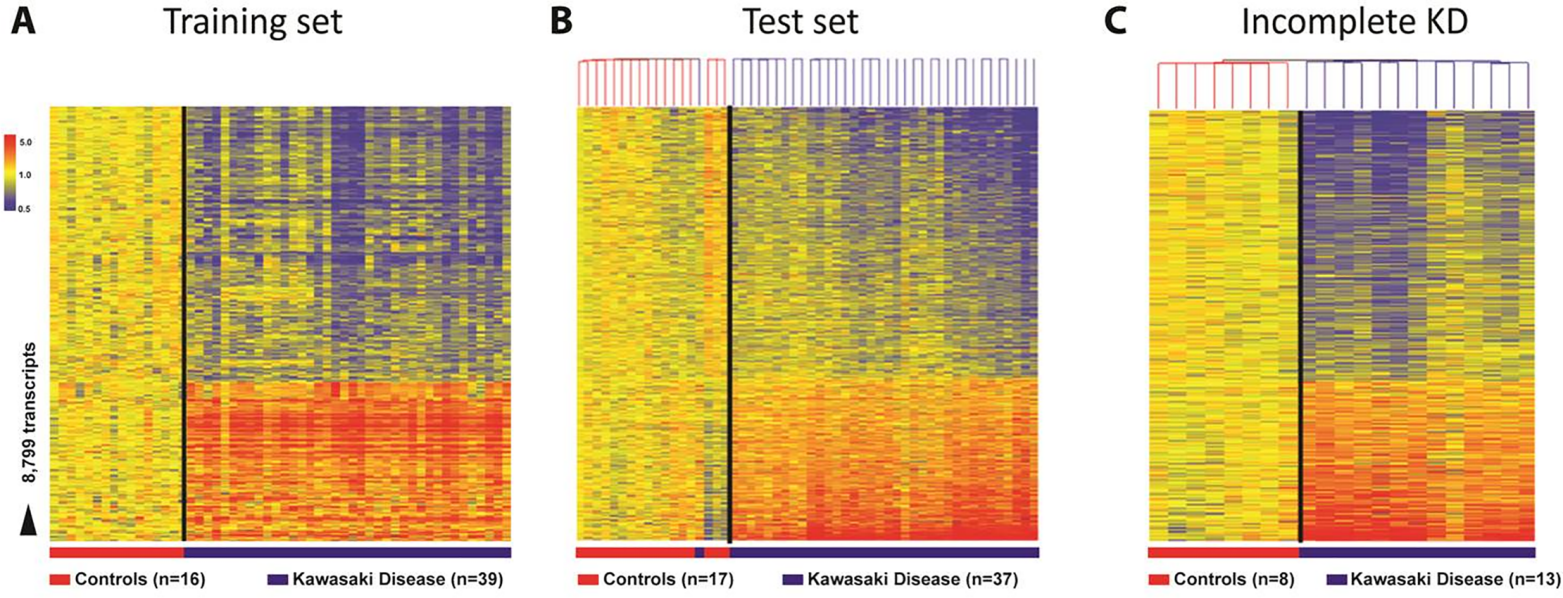
Biosignature of KD. **(**A) Statistical group comparisons between children with complete KD and healthy matched controls (HC) (Mann-Whitney test p<0.01, Benjamini-Hochberg multiple test correction and 1.25-fold change) yielded 8,799 significantly differentially expressed transcripts. Transcripts were organized by hierarchical clustering, where each row represents a single transcript and each column an individual participant. Normalized expression levels are indicated as overexpressed (red) or underexpressed (blue) compared to the median expression of healthy controls (yellow). (B) The 8,799 biosignature was applied to an independent test set of 37 children with complete KD and HC. Unsupervised hierarchical clustering of the KD signature grouped all complete KD patients in the test set together (blue bar) except for one patient who was on the ninth day of illness at the time of sample collection, and (C) all incomplete KD patients were grouped together. Black line indicates the cluster separation.

To define the transcriptional KD signature, children with complete KD (cKD) were divided randomly in two cohorts: training (discovery) and test (validation) sets. Demographic, clinical and laboratory data are listed in Table 1. Statistical group comparisons identified 8,799 differentially regulated transcripts between 39 KD patients and 16 healthy matched controls in the first group of patients (training set; Fig 2A). There were 5,517 (62.7%) transcripts underexpressed and 3,282 (37.3%) overexpressed. This signature was validated in a second cohort of 37 patients with complete KD and 20 healthy controls using unsupervised hierarchical clustering which grouped 36 of 37 KD patients together (Fig 2B). The only KD sample that clustered with the healthy control group was obtained in a 7-month-old male who was on day 9 of illness at the time of sample collection. A third, new group of children with incomplete KD (iKD; n = 13) and 8 matched HC was also analyzed. Unsupervised hierarchical clustering of the complete KD biosignature (8,799 transcripts) applied to this cohort correctly grouped together all patients with incomplete KD (Fig 2C).

**Table 1:** Demographic, clinical and laboratory data of study patients.

	Complete Kawasaki Disease (KD)						Incomplete KD		
	Training Set			Test set			Validation Set		
	Patients	Controls	p value	Patients	Controls	p value	Patients	Controls	p value
	(n = 39)	(n = 16)		(n = 37)	(n = 20)		(n = 13)	(n = 8)	
**Age** (years)	3.8 (2.1–5.1)	4.3 (1.9–9.8)	0.173	2.8 (1.4–5.9)	7.9 (3.2–12.7)	<0.001	4.1 (1.8–6.5)	3 (2.5–4.3)	0.587
**Male,** n (%)	23 (59)	6 (38)	0.234	21 (57)	9(45)	0.42	8(62)	4(50)	1
**Race/Ethnicity, n,** (%)			0.552			0.092			0.511
White	22 (56)	9(56)		16 (43)	14 (70)		9 (69)	4 (50)	
Black	9 (23)	2(13)		9 (24)	1 (5)		1 (8)	2 (25)	
Hispanic	3 (8)	1(6)		2 (5)	0(0)		1 (8)	0	
Other	5 (13)	4(25)		10 (27)	5(25)		2(15)	2 (25)	
**White blood cells** (1000/mL)	13.3 (10.5–20.2)	8 (4.4–9.5)	<0.001	13.5 (10.3–18.3)	6.65 (5.7–8.6)	<0.001	15.4 (10.5–19)	8.8 (8.4–9.2)	0.05
% Neutrophil	77 (60–84)	41 (32–50)	<0.001	63 (54–79)	49.000 (44–55)	p = 0.003	69 (45–87)	39 (28–50)	0.17
% Lymphocytes	15 (9–26%)	46 (31–51)	<0.001	27(12.5–40)	38 (34–44)	p = 0.014	18 (10–44)	50 (38–62)	0.267
% Monocytes	6 (3–8)	7 (6–10)	0.133	5(3–7)	8 (6–9)	p = 0.001	5 (1–10)	7 (6–7)	0.711
**KD Specific Data**									
**Fever at enrollment** (days)	6.0 [5.0–8]			5.0 [5.0–7.0]			7 (5–9]		0.209
**Treatment Non-Response** n, (%)	4/35 (11.4%)			5/33 (15.1%)			1/10 (10%)		0.893
**Coronary artery abnormalities,** n, (%)	5/33 (15%)			7/33 (21%)			0/10 (0%)		0.188
**Albumin** (gr/L)	3.3 (2.9–3.7)			3.7 (3.4–4.0)			3.0 (2.9–3.5)		0.003
**ESR** (mm/hr)	54 (40–74)			60 (47–74)			58 (41–96)		0.505
**CRP** (mg/dL)	13.7 (5.6–17.3)			10.8 (4.7–15.4)			10.9 (5.7–18.1)		0.726
**ALT** (mg/dL)	53 (23–103)			70 (28–127)			38 (25–114)		0.679
Pyuria* (WBC/mcrL)	13/34 (38%)			13/30 (43%)			4/10 (40%)		0.917

Continuous variables are reported as medians 25%-75% interquartile ranges (IQR). White blood cells (WBC). ESR: erythrocyte sedimentation rate; CRP: C-reactive protein; ALT: alanine aminotransferase

*Pyuria was defined as 10 WBCs/HPF or >8 cells/microliter for females; >20 cells/microliter for males

**Fig 3:**
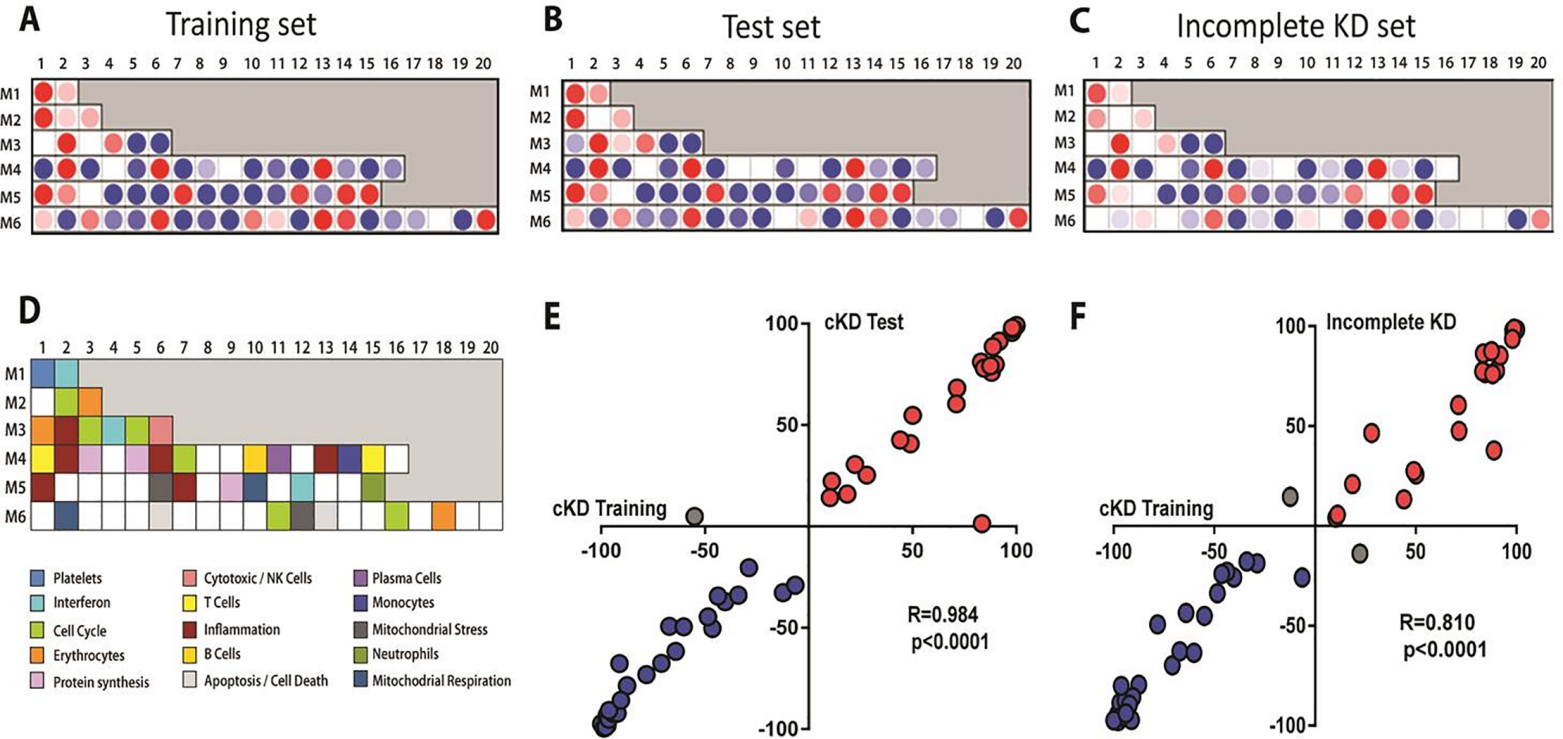
Modular transcriptional fingerprint for complete KD in the training, test and incomplete KD sets. Colored dots represent the percentage of significantly overexpressed (red) or underexpressed (blue) transcripts within a module in patients with complete KD compared to controls (see [D] for module map key). Blank modules indicate no significant differences between patients and controls. (A) Patients with complete KD demonstrated significant overexpression of modules related to inflammation (M3.2, M4.2, M4.6, M4.13, M5.1, 5.7), platelets (M1.1), neutrophils (M5.15). Conversely, genes related to adaptive immunity: T cells (M4.1, M4.15), B cells (M4.10), lymphoid lineage (M6.19), and cytotoxicity/NK cells (M3.6), were significantly underexpressed. (D) Key to the functional interpretation of each transcriptional module (M): module sets 1 to 6 are indicated on the y-axis, and module numbers within each set are indicated on the x-axis. Scatter plot showing modular correlations (Spearman's r) between training (x-axis) and the test (y-axis) sets (E) and incomplete KD set (F). The inflammation modules (M4.6, M4.2, 3.2) were the most highly correlated between the training and the test sets.

To characterize the biological significance of the KD biosignature, we then applied an analytical framework of 62 transcriptional modules [19, 22]. Module maps were derived independently for each of the complete KD cohorts (training set [Fig 3A], test set [Fig 3B]) as well as for children with incomplete KD (inKD) (Fig 3C). Children with complete KD demonstrated significant overexpression of modules related to inflammation, platelets, apoptosis, and neutrophils. Conversely, genes associated with B cells, T cells and cytotoxic/NK cells were significantly underexpressed. These findings were confirmed in the test (cKD) and validation (inKD) sets, as demonstrated by significant correlations of modular gene expression (r = 0.984 and 0.81, respectively Fig 3E and 3F).

### Transcriptional profiles in Kawasaki disease *versus* adenovirus and *Group A streptococcus* infections

**Fig 4:**
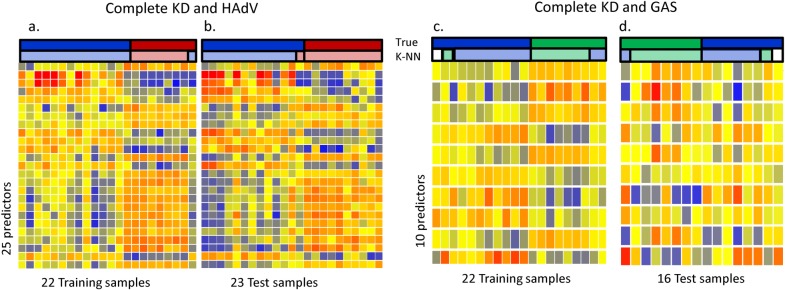
Transcriptional profiles in Kawasaki disease versus adenovirus and Group A streptococcus infections. Supervised learning KNN algorithm with 6 neighbors and a p value ratio cutoff of 0.5 was used to identify the top genes that best discriminated cKD from adenovirus (AdV) disease from (A) and GAS (B). Predicted class is indicated by the corresponding lighter-colored rectangles. **(A)** The KNN algorithm identified 25 classifier genes that best discriminated AdV from KD. Leave one out cross validation of the training set (A) and test set (B) correctly classified 43 of 45 patients with 96% accuracy (complete KD [n = 26; blue); AdV [n = 19, red]. **(B)** Using the same strategy we identified 10 genes that best discriminated GAS from cKD. (C and D). Leave-one-out cross-validation of the 10 classifiers in the training set (C) and test set (D) correctly classified 28 of the 36 samples (complete KD [n = 19; blue]; GAS [n = 17, green] with 78% accuracy; one patient in the training set and test sets could not be predicted.

To determine whether the systemic host immune response in children with KD was different compared with those induced by adenovirus and GAS infections (two common mimicking illnesses) we applied a K-nearest neighbor (K-NN) class prediction algorithm (Fig 4). Confirmed adenovirus infection was defined as a positive nasopharyngeal culture or PCR and considered clinically consistent with adenovirus disease. Children with GAS infection, included those with a positive culture from a sterile site (n = 14), or scarlet fever (n = 3).

The KNN algorithm identified 25 classifier genes that best discriminated KD from adenovirus infection in two independent groups of patients (Fig 4). The 25 classifier genes correctly classified 21 of 22 patient samples in the training set. This was validated in an independent test set, for which the classifier genes correctly categorized 21 of 22 patients. Overall, the 25 classifier genes discriminated KD from adenovirus infection with 92% (95% CI [69%-99.7%]) sensitivity and 100% [69%-100%] specificity in the test set (Table 2A). We also applied the KNN algorithm to discriminate KD from GAS and identified 10 classifier genes that best discriminated KD from GAS infection also in two independent cohorts of patients. The 10-classifier genes correctly classified 16 of 20 samples in the training set, and 13 of 16 in the test set. The 10 classifier genes discriminated KD from GAS infection with 75% (95% CI [34%-96%]) sensitivity and 87% [47%-99%] specificity in the test set (Table 2B). S1 Table includes a description of the respective 25 and 10 classifier genes.

**Table 2:** Performance characteristics of 25 and 10 classifier genes to discriminate complete KD versus adenovirus (A) and GAS infections (B).

**(A)**	**Training** **(n = 21)**			**Test** **(n = 22)**		
	**KD** **(n = 13)**	**HAdV** **(n = 9)**	**P-value**	**KD** **(n = 12)**	**HAdV** **(n = 10)**	**P-value**
**Age (months)**	2.6 (2.2–4.6)	4.9 (1.9–6.8)	0.462	3.4 (2.1–5.6)	4.3 (1.9–5.4)	0.974
**Sex (M:F)**	10M: 3F	7M:2F	1.000	7M:5F	3M:7F	0.231
***Classification***						
**Correct (%)**	13	8	NA	11	10	NA
**Incorrect (%)**	0	1	NA	1	0	NA
**Not classified (%)**	0	0	NA	0	0	NA
**Sensitivity for KD (95% CI)**	100% (75–100%)			92% (69–99.7%)		
**Specificity for KD (95% CI)**	88% (51–99%)			100% (69–100%)		
**(B)**	**Training** **(n = 20)**			**Test** **(n = 16)**		
	**KD** **(n = 11)**	**GAS** **(n = 9)**	**P-value**	**KD** **(n = 18)**	**GAS** **(n = 8)**	**P-value**
**Age (months)**	5.2 (4.4–6.7)	6.5 4.4–10.4)	0.494	3.8 (2.6–6.4)	3.8 (1.4–5.3)	0.645
**Sex (M:F)**	6M: 5F	7M:2F	0.374	4M:4F	3M:5F	1.000
***Classification***						
**Correct (%)**	9	7	NA	6	7	NA
**Incorrect (%)**	1	2	NA	1	1	NA
**Not classified (%)**	1	0	NA	1	0	NA
**Sensitivity for KD (95% CI)**	81% (48–97%)			75% (34–96%)		
**Specificity for KD (95% CI)**	77% (40–97%)			87% (47–99%)		

**Fig 5:**
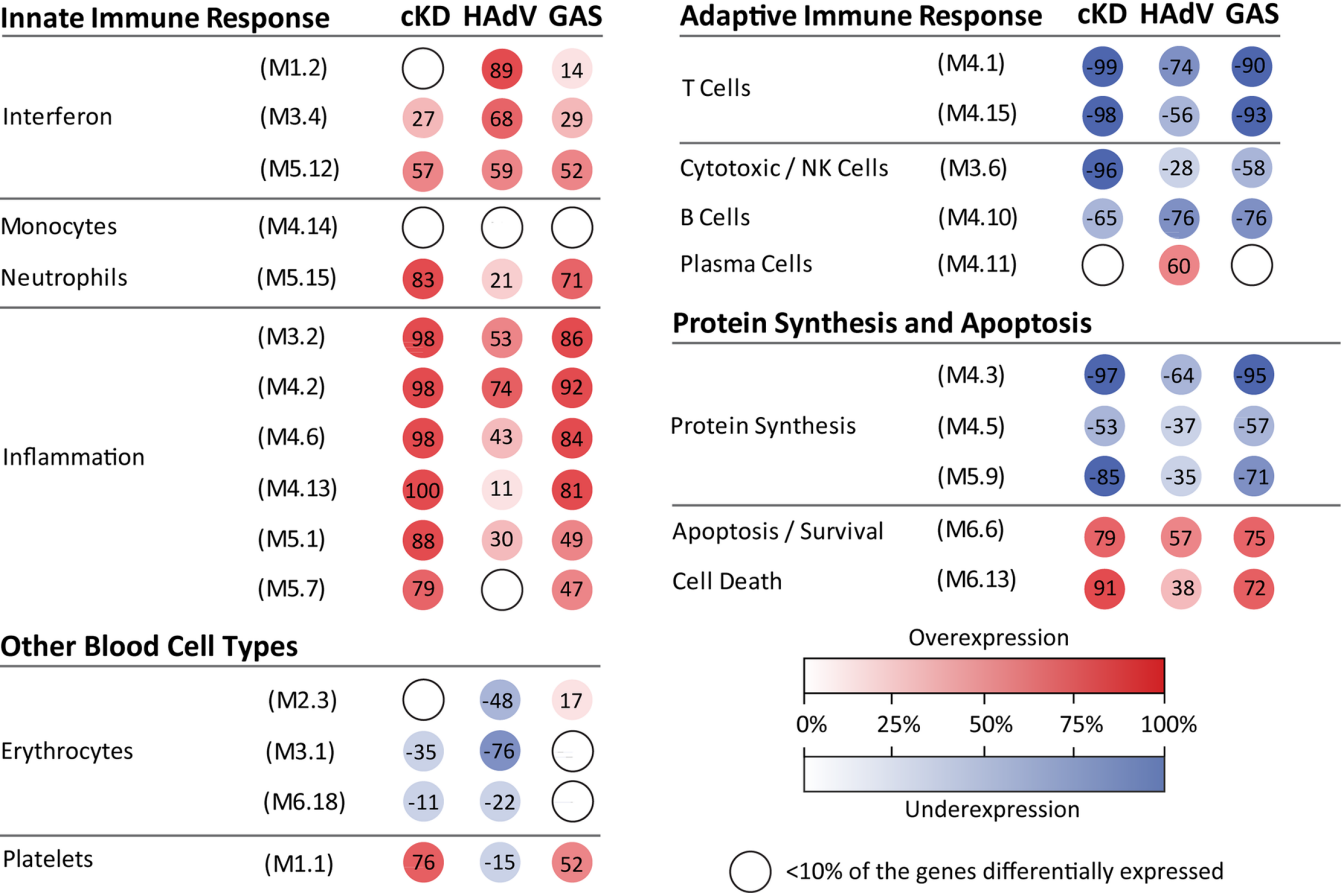
Modular fingerprints of patients with KD, GAS and adenovirus infections. Modular transcriptional fingerprints were derived and compared in children with cKD (n = 39), GAS (n = 17) and adenovirus (n = 19) infection. Colored spots represent the percentage of significantly overexpressed (red) or underexpressed (blue) transcripts within a module in patients with a disease state compared with age- and sex-matched healthy controls (16 HC for KD comparisons and 10 HC for GAS and AdV respectively).

To further define the differences in immune profiles among these three conditions, we compared the modular fingerprints from children with KD (training set), adenovirus and GAS infections (Fig 5). Each disease group was compared with an age- and sex-matched healthy control group as reference. Although several modules showed similar expression patterns among the three conditions, the magnitude of over- or underexpression was significantly greater in children with KD. Specifically, children with KD demonstrated greater overexpression of several inflammation (M4.6, M4.13, M5.1 and M5.7) and neutrophil (M5.15) related modules while cytotoxic T cells/NK cell related genes (M3.6) were greatly underexpressed compared with both HAdV and GAS infections. Interestingly, the interferon (M1.2) and plasma cell (M4.11) modules were markedly overexpressed in children with adenovirus infection but not in KD or GAS infections.

### Molecular distance to health scores and clinical outcomes

**Fig 6:**
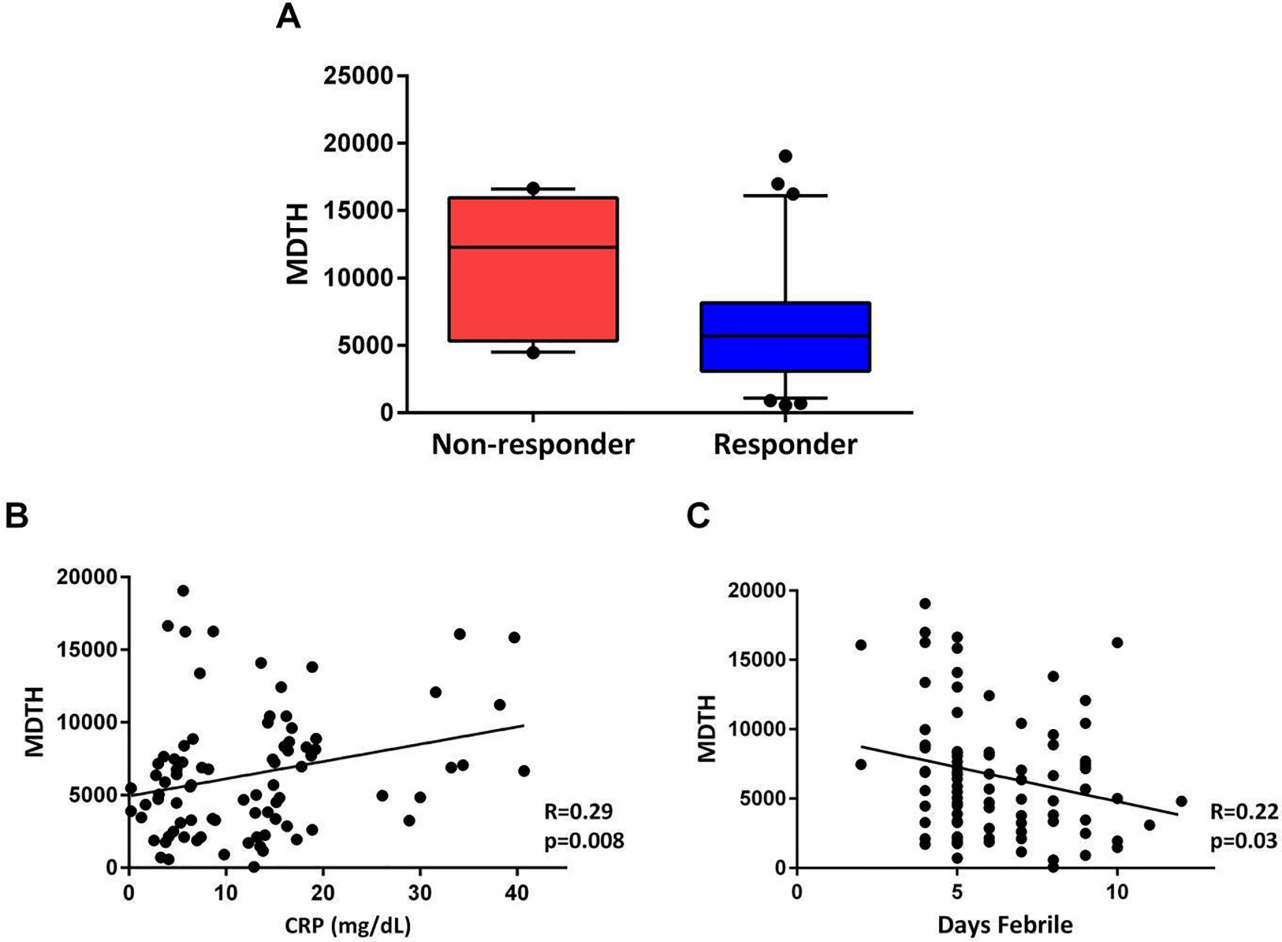
Molecular distance to health scores (MDTH) in children with KD. MDTH scores are a metric that converts the global transcriptional perturbation of each sample into an objective score indicating the degree of transcriptional perturbation of each individual patient compared with a healthy control baseline. (A) MDTH scores were significantly higher in patients who failed to respond to initial treatment with IVIG, (12,290 vs. 5,019; p = 0.009) (B) MDTH scores were correlated with pre-treatment, baseline C-reactive protein serum concentrations [n = 84], (C) and inversely correlated with days of fever at the time of sample acquisition, (Spearman’s correlation coefficient).

We next investigated whether a genomic score (molecular distance to health [MDTH] score) could help predicting: a) the response to initial treatment with IVIG, and b) the development of coronary artery abnormalities in children with complete and incomplete KD. Of 77 KD patients in whom complete clinical data were available, 10 (13%) did not respond to therapy. By the 5–8 week visit, 12 (15.5%) subjects had abnormal dimensions for the coronary arteries on at least one echocardiogram. MDTH scores from samples obtained at enrollment and before IVIG therapy were significantly higher in children who remained febrile 36 hours after initial treatment with IVIG and required additional therapy compared with those who responded (Fig 6). There was no correlation of MDTH with the development of CAA. The MDTH scores at enrollment correlated weakly with C-reactive protein (CRP) serum concentrations (r = 0.29, p = 0.008) and inversely correlated with days of fever (r = -0.22, p = 0.03).

### Longitudinal assessment of molecular distance to health scores

**Fig 7:**
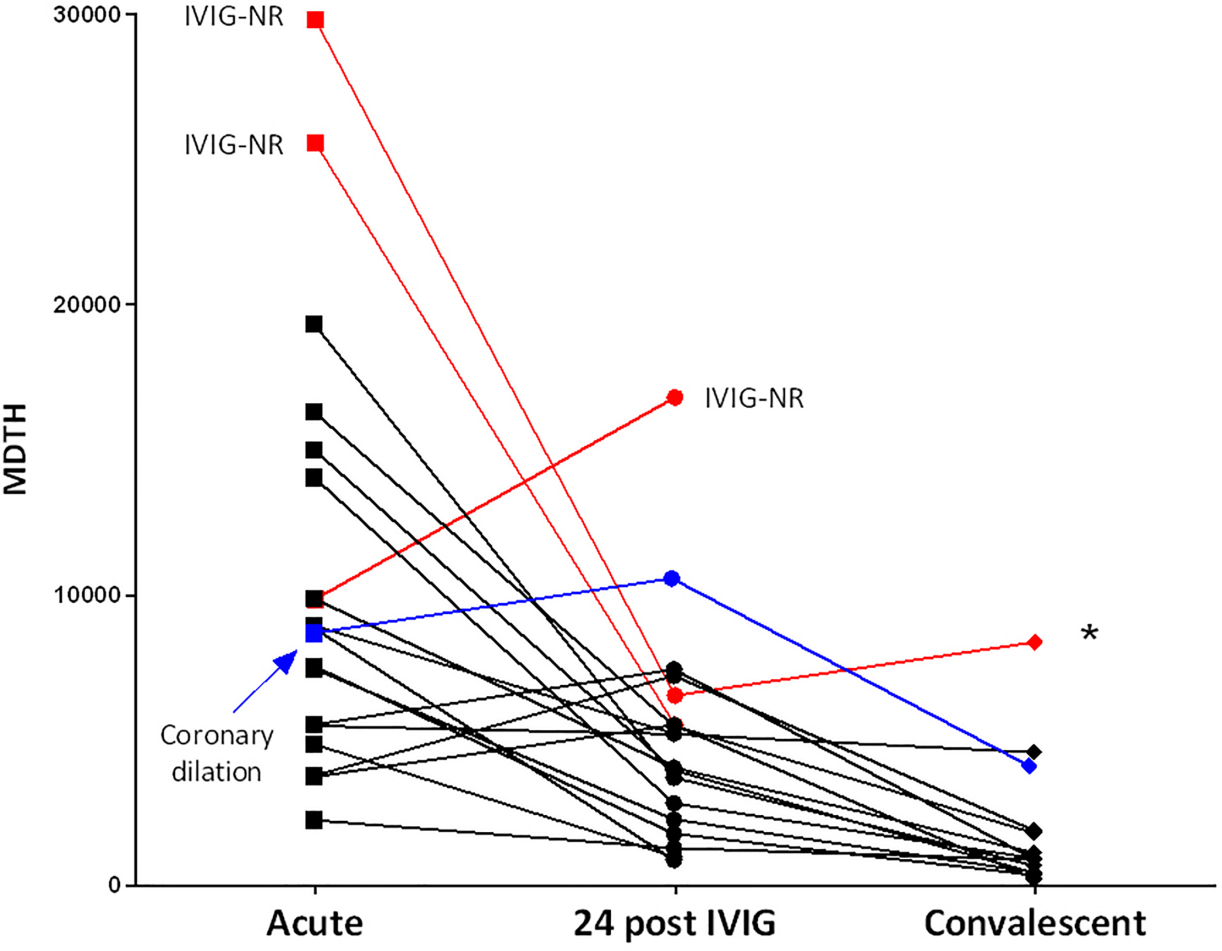
MDTH scores measured over time. MDTH scores were analyzed in 18 patients with available sequential samples: acute (pre-treatment), 24 hours after IVIG (24 post IVIG), and convalescent (5–8 weeks after treatment). The three patients that failed to respond to the first dose of IVIG (IVIG-NR) are shown in red color, and the patient with coronary artery dilation, is shown in blue color. One subject, marked with an asterisk (*), was a non-responder to IVIG, required a corticosteroid taper and had residual arthralgias on the follow-up visit during convalescence (in red).

Sequential samples were available from 18 patients before and 24 hours after completion of IVIG treatment. Of these 18 patients, three did not respond to initial IVIG treatment and one developed coronary artery dilatation. In addition, 13 out of these 18 patients had samples available at 5–8 weeks post treatment (convalescent). Longitudinal analysis showed a significant decrease in MDTH scores in 14 of 18 patients 24 hours after completion of IVIG (Fig 7). Of the four patients in whom MDTH increased at 24h post-IVIG, two had slight increased values and responded to IVIG treatment, another developed coronary artery dilation, and the fourth child had a significant increase in MDTH values and did not respond to the first dose of IVIG. By 5–8 weeks after IVIG, MDTH values significantly decreased in the majority of patients (85% or 11 of 13 patients). There were two exceptions, one patient was an IVIG-non responder and required a corticosteroid medication taper. He had residual arthralgias on the day of sample collection. The other patient was doing clinically well on the follow-up evaluation, but had a documented visit to the urgent care one week earlier and was diagnosed with an upper respiratory tract infection.

### Molecular distance to health scores independently predicts response to intravenous immunoglobulin therapy at enrollment

**Fig 8:**
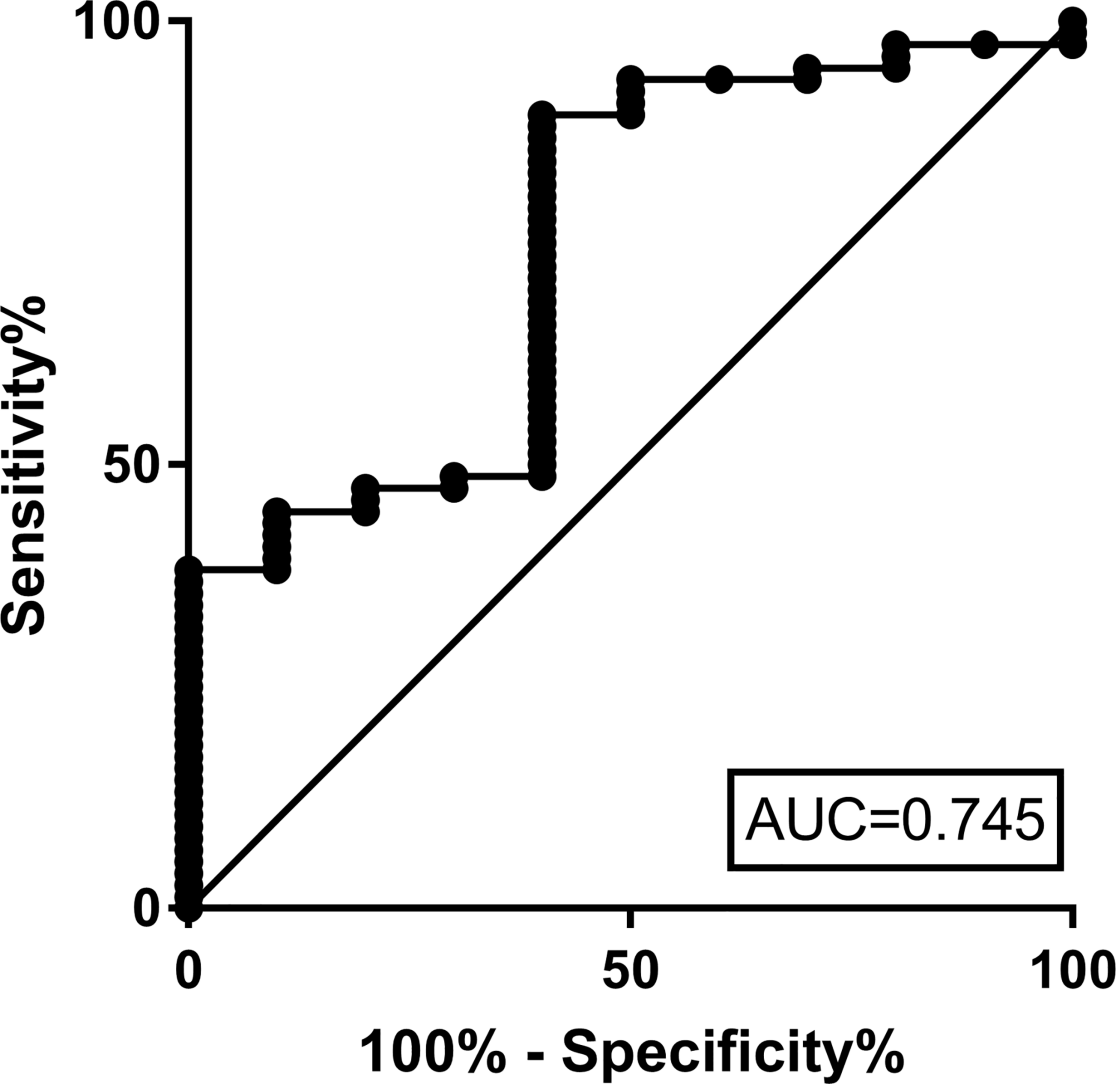
Area under the ROC curve (AUC) of MDTH scores to predict response to IVIG therapy. The optimal threshold for MDTH defined using Youden’s J statistic, which maximizes sensitivity and specificity is 10,428. When MDTH is dichotomized as “low” or “high” based on this threshold, low MDTH values have 88% sensitivity and 60% specificity for classifying patients as responders, and AUC remains good at 0.741.

To determine the ability of MDTH to discriminate between response and non-response to IVIG, we constructed the receiver operating characteristic (ROC) curve (AUC) using the pre-treatment MDTH values for KD patients that did and did not responded to the first dose of IVIG. The optimal threshold for MDTH was identified at 10,428 which gave an AUC of 0.74 [95% CI (0.57–0.90)]. Low values of MDTH (< 10,428) using this threshold had an 88% sensitivity and 60% specificity for classifying KD patients responders to treatment with IVIG (Fig 8).

Last, we performed multivariable logistic regression analysis to identify which factor(s) alone or in combination best predicted response to IVIG therapy. After adjusting for gender, days of fever, type of KD (incomplete vs. complete), albumin, and CRP concentrations, only high MDTH values (OR = 1.72 [1.18–2.48]) and younger age (OR = 0.94 [0.89–0.99]), independently predicted lack of response to IVIG therapy (Table 3).

**Table 3:** Multivariable logistic regression for the outcome of non-response to treatment.

	Non-Response to Treatment		Coronary Artery Abnormalities	
	Odds Ratio, (95% CI)	*p* value	Odds Ratio, (95% CI)	p value
**Gender: Male**	0.224 (0.023–2.17)	0.196	1.226 (0.322–4.68)	0.765
**Age (months)**	0.947 (0.899–0.999)	**0.046**	1.000 (0.979–1.021)	0.990
**Days of Fever at Sample collection**	1.126 (0.613–2.069)	0.702	0.952 (0.675–1.343)	0.780
**MDTH score/per 1000**	1.716 (1.186–2.484)	**0.004**	1.012 (0.84–1.22)	0.901
**CRP (mg/dL)**	0.994 (0.877–1.128)	0.931	1.049 (0.977–1.127)	0.190
**Albumin (mg/dL)**	0.932 (0.122–7.106)	0.946	0.740 (0.226–2.428)	0.620
**Complete KD**	0.054 (<0.001–3.377)	0.166	0.365 (0.035–3.846)	0.401

Gender male (reference is female); MDTH: molecular distance to health; CRP: C-reactive protein; Complete KD (incomplete KD is the reference)

## Discussion

Using RNA transcriptional profiling, we identified a distinct biosignature in children with complete KD that was validated in two independent patient cohorts, one comprised of children with complete KD and a second of children with incomplete KD. We also identified classifier genes that discriminated KD patients from those with adenovirus infection quite precisely and less so from patients with GAS, two common conditions that mimic KD. Lastly, we defined a genomic MDTH score at presentation that was associated with resistance to IVIG therapy. Overall, this study confirms the potential application of transcriptional profiling for the diagnosis of KD and the utility of genomic scores to predict response to therapy in children with KD before IVIG treatment.

Previous studies have also described overexpression of innate immunity transcripts and down regulation of T and B cell receptors and NK cell signaling in patients with KD compared with febrile control patients [23–25]. In the present study, we identified a distinct and reproducible KD biosignature that was validated in a second independent cohort of KD patients, and more importantly in a third group of children presenting with incomplete KD, supporting the value of the current clinical criteria for the diagnosis of KD.

Host expression patterns based on RNA transcriptional profiles have been used to distinguish bacterial from viral infection in young febrile infants and children [13, 18, 26], and also to discriminate patients with KD from those with adenovirus infection [15]. Although appealing, it is challenging to use host expression patterns in a disease that lacks diagnostic confirmation, especially in those with the less common incomplete presentation of the disease. One example of a similar scenario in which diagnostic confirmation is challenging is pediatric tuberculosis. In a recent study, a genomic signature was identified first in children with confirmed TB and was then applied to patients with “probable” or “possible” disease with 82% sensitivity and 84% specificity. This approach emphasizes the utility of genomic signatures when initially applied to patients with clearly defined illness, followed by application to other cohorts with less defined clinical diagnosis [14, 27]. We followed a similar approach as we first derived the KD biosignature from children with complete KD, and then applied it to children with less clearly defined illness, incomplete KD. Indeed, it was remarkable to observe that patients with incomplete KD had a profile almost identical to those with cKD. The peripheral blood KD biosignature, for both complete and incomplete KD, was characterized by marked overexpression of inflammation, apoptosis, platelets and neutrophil genes, and with marked underexpression of cytotoxic T cell and NK cell related genes. This is in contrast to the findings of gene expression at the level of coronary tissue, where cytotoxic T cells have been shown to be upregulated [28]. These findings have significant implications at they suggest that complete and incomplete KD share similar immunopathogenesis. In addition, these findings emphasize the importance of early identification and treatment of patients with incomplete KD, as their immune response is similarly dysregulated. It also adds to the validation of the AHA clinical criteria to identify and diagnose patients with incomplete KD [29, 30].

Distinguishing patients with adenovirus infection from KD can be difficult as adenovirus infections are common in this age group, often result in elevated inflammatory markers, and because adenovirus can be detected incidentally in children with KD [31, 32]. The present study showed that transcriptional profiling can help discriminate adenovirus from KD with high accuracy, confirming previous reports [15]. The modular analysis provided additional detail of the biological differences between the two conditions. KD patients had overexpression of two of the three interferon modules. The overexpression of module 1.2, more reflective of type I interferon, however, was absent in children with KD [33]. In agreement with these findings, previous studies have reported that KD patients had reduced expression of type I interferon transcripts compared with those with adenovirus infection [15]. Using transcriptional profiles to differentiate KD from GAS infection proved to be more difficult, though distinguishing KD from GAS disease in the clinical setting is less problematic as the organism can be isolated from the pharynx or blood in most cases of invasive disease. The majority of patients in this group had GAS identified from a normally sterile body site, so were not the typical patient in whom KD is considered in the differential diagnosis, and only 3 patients had scarlet fever, which would be more typically associated in the differential diagnosis of KD. Earlier studies have described similarities in gene expression profiles between bacterial infections and various autoimmune processes [11], likely reflecting downstream inflammatory cascades that are shared between these conditions.

Kawasaki disease is a dynamic process reflecting acute and subacute stages and our patients were enrolled at varying days of fever. Increasing days of fever at the time of the sample collection was inversely correlated with the MDTH score. Further investigation with a substantial number of patients treated later in the course of KD illness will be extremely important, as these children may no longer exhibit the biosignature observed in children diagnosed earlier. Thus, we believe that duration of fever is likely a crucial factor to consider when interpreting host expression data, and we accounted for such factor when we conducted our multivariable models.

Japanese clinical scoring systems have been successful in identifying high-risk patients to facilitate use of adjuvant therapies such as corticosteroids for primary treatment of KD, which have been associated with decrease coronary artery aneurism development in these children [6]. Those scoring systems have not been validated in ethnically diverse non-Japanese populations [34, 35]. Identification of potential non-responders before IVIG treatment using genomic scores (MDTH) in an ethnically diverse population may potentially allow early use of adjunctive therapies in selected patients, which in turn could be associated with improved coronary outcomes [6, 30]. Previous studies have utilized whole blood transcriptional patterns to identify specific transcripts that were over-expressed in patients with eventual IVIG resistance [36, 37], as well as serum biomarkers and cytokines such as G-CSF and sTNFR1 that were correlated with lack of response to therapy. [38, 39][40]. We utilized a slightly different approach with the MDTH score, and instead of focusing on a specific marker, we evaluated the overall molecular perturbation in KD patients in relation to the healthy control baseline. The MDTH scores from KD patients before IVIG treatment were significantly higher in children who were eventually IVIG resistant. Furthermore, among the laboratory variables evaluated, only high MDTH scores independently and incrementally predicted lack of response to therapy.

Our study has limitations. Because the limited number of patients with coronary abnormalities in our cohort, we were unable to identify any correlation between pre-treatment MDTH scores and development of coronary abnormalities (CAA). Profiles were derived from peripheral whole blood and may not reflect the pathophysiology of arterial damage, the most relevant site of inflammation in KD. On the other hand, KD is a systemic process and this type of analytical strategy has been used successfully to identify biomarkers and characterize the immunopathogenesis of other childhood vasculitides [11, 33].

In summary, this study provides further evidence of the marked dysregulation of the systemic inflammatory response induced by KD disease and confirms the value of transcriptional profiling as a promising strategy to determine eventual lack of response to IVIG, after adjusting for other variables in this cohort. As new and potentially more rapid technologies become available, transcriptional profiling in the clinical setting has the potential for use to improve the risk stratification of KD patients.

## Supporting information

S1 Table 10 classifier genes that best discriminated between Kawasaki disease and Group A streptococcus infection and the 25 classifier genes that best discriminated between Kawasaki disease and adenovirus infection. The systematic, common and genebank names are included in the first three columns. Function of those genes and analytical groups (KD vs GAS or KD vs. HAdV) are included in the 4^th^ and 5^th^ columns.
(DOCX)

## Abbreviations

KD: Kawasaki Disease
IVIG: intravenous immunoglobulin
RNA: ribonucleic acid
MTC: multiple test comparisons
MDTH: molecular distance to health
NK: natural killer
