# Supplementary material for: Whole blood transcriptional profiles as a prognostic tool in complete and incomplete Kawasaki Disease
Source: PLoS One. 2018 May 29;13(5):e0197858. doi: 10.1371/journal.pone.0197858 (PMC5973615; doi:10.1371/journal.pone.0197858)
Supplement: S1 Table — The systematic, common and genebank names are included in the first three columns. Function of those genes and analytical groups (KD vs GAS or KD vs. HAdV) are included in the 4th and 5th columns. (DOCX) [file pone.0197858.s001.docx]

**S1 Table. Classifier Genes that best discriminate Kawasaki Disease (KD) versus adenovirus (HAdV) and Group A streptococcus (GAS) infections**

| Systematic | Common | Genbank | Function | Discrimination |
| --- | --- | --- | --- | --- |
| ILMN_1789830 | CFLAR | NM_003879.3 | Apoptosis regulator protein | KD vs. GAS |
| ILMN_1673252 | AIMP2 | NM_006303.3 | Pro-apoptotic factor | KD vs. GAS |
| ILMN_2051232 | SDHA | NM_004168.1 | Subunit of mitochondrial resp chain | KD vs. GAS |
| ILMN_1769782 | LAX1 | NM_017773.2 | Negatively regulates T and B cell signaling | KD vs. GAS |
| ILMN_1758087 | TAOK1 | NM_020791.1 | Regulates apoptosis, DNA damage repair | KD vs. GAS |
| ILMN_1813207 | MRPS9 | NM_182640.1 | Mitochondrial protein synthesis | KD vs. GAS |
| ILMN_1681301 | AIM2 | NM_004833.1 | Cell proliferation. Interferon-gamma induces expression of AIM2 | KD vs. GAS |
| ILMN_1758938 | SLC31A2 | NM_001860.2 | Transport of glucose, sugars | KD vs. GAS |
| ILMN_1736510 | FOXN2 | NM_002158.3 | Transcriptional regulator of T cell leukemia virus | KD vs. GAS |
| ILMN_3191695 | LOC100128266 | XR_037888.1 | unknown | KD vs. GAS |
| ILMN_1773742 | DNAJB9 | NM_012328.1 | Protects stressed cells from apoptosis | KD vs. HAdV |
| ILMN_1733675 | MPP1 | NM_002436.2 | Regulates cell proliferation | KD vs. HAdV |
| ILMN_1768127 | EBNA1BP2 | NM_006824.1 | Segregates EBV episomes during cell division | KD vs. HAdV |
| ILMN_1654875 | CLC | NM_001828.4 | Lysophospholipase expressed in eosinophils, basophils, may have role in inflammation | KD vs. HAdV |
| ILMN_3257030 | FTHL16 | XR_016025.2 | unknown | KD vs. HAdV |
| ILMN_1719392 | FH | NM_000143.2 | Enzymatic component of Krebs cycle | KD vs. HAdV |
| ILMN_1701512 | KIAA0391 | NM_014672.2 | mitochondrial ribonuclease | KD vs. HAdV |
| ILMN_3241091 | LOC100130886 | XM_001714477.1 | unknown | KD vs. HAdV |
| ILMN_1667418 | LOC283953 | XM_208930.4 | unknown | KD vs. HAdV |
| ILMN_1801043 | GSN | NM_198252.2 | Regulation of actin cytoskeleton | KD vs. HAdV |
| ILMN_1680239 | NUDT9 | NM_198038.1 | Cellular metabolism | KD vs. HAdV |
| ILMN_1744147 | CEBPZ | NM_005760.2 | Direct p53 effector | KD vs. HAdV |
| ILMN_1749432 | MRPL32 | NM_031903.1 | Mitochondrial protein synthesis | KD vs. HAdV |
| ILMN_1759952 | PSMA5 | NM_002790.2 | processing of class I MHC peptides | KD vs. HAdV |
| ILMN_1670901 | COX10 | NM_001303.2 | terminal component of the mitochondrial respiratory chain | KD vs. HAdV |
| ILMN_2397954 | PARP3 | NM_005485.3 | DNA repair, regulation of apoptosis | KD vs. HAdV |
| ILMN_2160929 | FEN1 | NM_004111.4 | Repair of breaks in DNA | KD vs. HAdV |
| ILMN_2286514 | GTPBP8 | NM_014170.2 | protein coding | KD vs. HAdV |
| ILMN_1759008 | ZNF689 | NM_138447.1 | Transcriptional regulation | KD vs. HAdV |
| ILMN_1689652 | RNMTL1 | NM_018146.2 | Unknown | KD vs. HAdV |
| ILMN_1686109 | CCL23 | NM_145898.1 | T cell and monocyte chemokine | KD vs. HAdV |
| ILMN_1759117 | XK | NM_021083.2 | Membrane transport protein | KD vs. HAdV |
| ILMN_1795991 | C22ORF28 | NM_014306.3 | tRNA-splicing ligase complex | KD vs. HAdV |
| ILMN_1703132 | LYRM2 | NM_020466.4 | unknown | KD vs. HAdV |
| ILMN_3227315 | LOC729009 | XR_042330.1 | unknown | KD vs. HAdV |
